# A Review of Epidemiologic Studies of the Health of Gulf War Women Veterans

**DOI:** 10.15436/2378-6841.17.1551

**Published:** 2017-08-24

**Authors:** Steven S. Coughlin, Maxine Krengel, Kimberly Sullivan, Penny F. Pierce, Vahé Heboyan, Lt Col Candy Wilson

**Affiliations:** 1 Department of Clinical and Digital Health Sciences, Augusta University, Augusta, GA; 2 Charlie Norwood VA Medical Center, Augusta, GA; 3 Boston Veterans Administration Healthcare System, Boston, MA; 4 Boston University School of Public Health, Boston, MA; 5 Uniformed Services University Graduate School of Nursing, Bethesda, MA; 6 Malcolm Grow Medical Clinics and Surgery Center at Joint Base-Andrews, MD

**Keywords:** Gulf War syndrome, Gulf War veterans, Symptoms, Epidemiology, Women’s health

## Abstract

**Introduction:**

In the 25 years since the 1990–1991 Gulf War (GW), studies have evaluated Gulf War Illness (GWI), sometimes referred to as medically unexplained multi symptom illness, and other medical and neurological conditions in women GW veterans.

**Materials and Methods:**

In this article, we review epidemiologic studies of the health of women who served in the 1990–1991 GW based upon bibliographic searches in PubMed and CINAHL with relevant search terms through September 2015.

**Results:**

A total of 56 articles were identified in the bibliographic searches. By screening abstracts or full-text articles, a total of 21 relevant studies were identified. Results from some studies, but not all, suggest that GWI is more common in women GW veterans than their male counterparts. Few studies of GW veterans focused on women’s health. A small number of studies suggested excess rates of woman’s health problems, e.g., breast cysts, abnormal Papanicolaou (Pap) smears, yeast infections, and bladder infections. Several studies have identified significantly elevated rates of birth defects and adverse reproductive outcomes among GW veterans. However, findings have varied with different study designs and sample sizes, with some studies showing elevated risks of stillbirths, miscarriages, and/or birth defects and others have not. In some studies, participants reported increased risks of ectopic pregnancies and spontaneous abortions.

**Conclusion:**

Further research is needed to provide a comprehensive picture of the health of women GW veterans and to examine a broad range of women’s health issues including adverse reproductive outcomes. Some deployment-related health problems only become apparent decades later and other conditions may worsen or improve over time. Assessments are needed of current health status, changes in health symptoms and conditions over time, and possible differences in health outcomes associated with specific experiences and exposures during the war. Future studies would be strengthened by assessing GWI symptom patterns that may be specific to women veterans, examine diagnosed medical conditions among women veterans, and evaluate changes in women’s health over time, including changes potentially associated with menopause and age.

## Introduction

Women comprised almost 7 percent of the nearly 700,000 military personnel who served in the 1991 Gulf War and represented the largest proportion of U.S. women serving in a war zone in U.S. military history to that point in time. In addition, expanded military roles for women increased their exposure to more intense levels of combat and to toxicant exposures (Bond, 2004). Woman GW veterans, therefore, may have health issues specific to their cohort that are more frequent than among other cohorts of women veterans or among non-veteran women. Several studies have examined rates of Gulf War Illness (GWI) and rates of psychiatric illnesses by gender in GW veterans. GWI illness is sometimes referred to as medically unexplained multisymptom illness or chronic multisymptom illness (Wolfe, et al. 2002; Coughlin, et al. 2013).

Some results from (Wolfe, et al. 2002; Coughlin, et al. 2013) but not all (Unwin, et al. 2002) suggest that GWI is more common in women GW veterans than their male counterparts and additional studies have found no gender difference in the prevalence of GWI. A small number of studies suggested excess rates of woman’s health problems, e.g., breast cysts, abnormal Papanicolaou (Pap) smears, yeast infections, and bladder infections (Pierce, 1997; Unwin, et al. 2002; Pierce, 2005). However, in the 25 years since the war, few studies have evaluated GWI and other medical and neurological conditions specifically in women GW veterans (Pierce, 1997). In addition to the lack of studies specific to gender, the original research that was conducted was more than 10 years ago and to our knowledge, follow-up studies have not been completed.

In this article, we review published epidemiologic studies on the health of GW women and identify gaps in our current understanding of the health of women who were deployed to the GW or who served elsewhere during that era. We also summarize published studies of adverse reproductive outcomes such as ectopic pregnancies, spontaneous abortions, stillbirths, and birth defects. Finally, we offer several recommendations for further research on the health of women GW veterans.

## Methods

**Figure 1:**
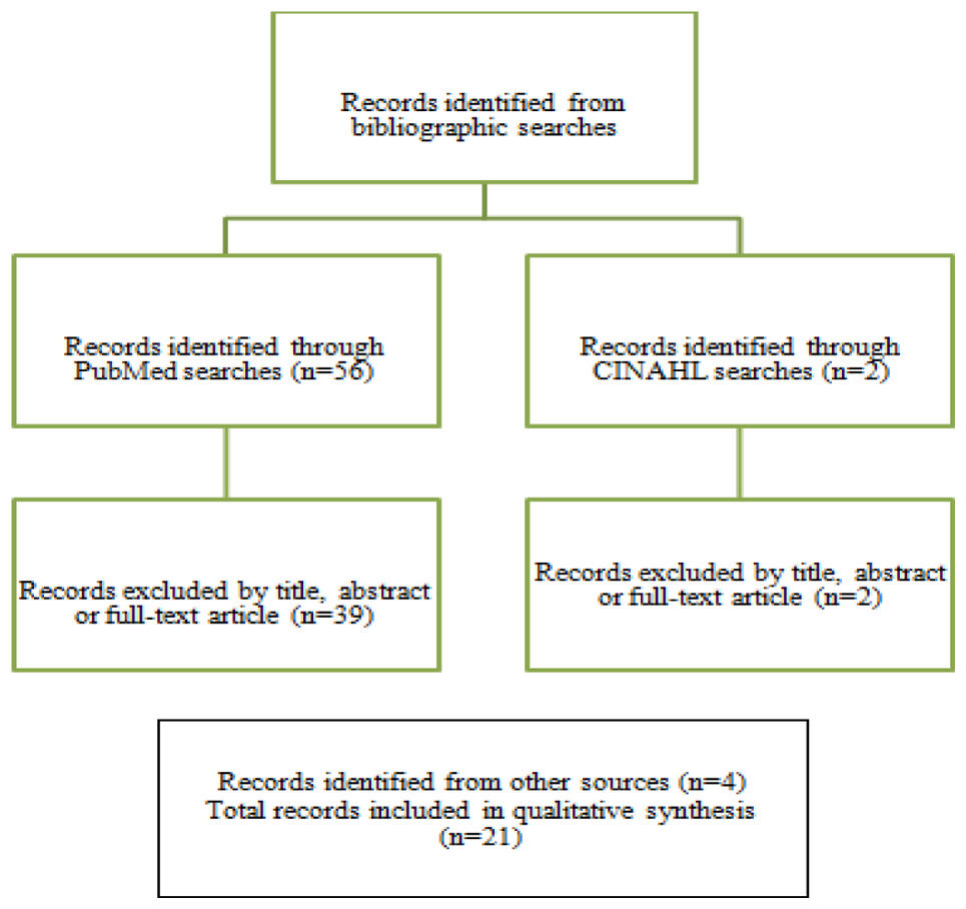
Summary of search and exclusion process.

The present review is based upon bibliographic searches in PubMed and CINAHL and relevant search terms. Articles published in English from 1990 through September 2015 were identified using the following medical subject heading (MeSH) search terms and Boolean algebra commands: (Gulf War) and (veterans health) and ((women’s health) or (women)). The searches were not limited to words appearing in the title of an article. Information obtained from bibliographic searches (title and topic of article, information in abstract, and key words) was used to determine whether to retain each article identified in this way. In addition, we reviewed the references of reports prepared by the Institute of Medicine and the Research Advisory Committee on Gulf War Illness Research and published review articles. A total of 56 article citations were identified in the bibliographic searches as detailed in Figure 1. After screening the abstracts or full texts of these articles, and examining the references of review articles and reports, 21 studies were identified for further review. This included one study that focused specifically on women GW veterans (Pierce, 1997; Pierce, 2005); a study of women veterans from more than one era, in which results were reported separately by era (Washington, et al. 2013); 10 studies of both male and female GW veterans in which at least some results were reported separately for women (CDC, 1995; Fukuda, et al. 1998; Steele, 2000; Wolfe, et al. 2002; Kang, et al. 2005; Coughlin, et al. 2013; Smith, et al. 2013; Provenzale D, 2015); and 9 studies of adverse reproductive outcomes and birth defects (Cowan, et al. 1997; Araneta, et al. 2000; Kang, et al. 2001; Araneta, et al. 2003; Araneta, et al. 2004; Doyle, et al. 2004; Wells, et al. 2006; Kelsall, et al. 2007; Verret, et al. 2008). Several studies were also identified of mortality and cancer incidence among male and female GW veterans (Kang, et al. 1996; Macfarlane, et al. 2000; Kang, et al. 2001; Macfarlane, et al. 2003; Macfarlane, et al. 2005; Lincoln, et al. 2007). The present review extends upon the work of earlier authors (Pierce, 1997; Unwin, et al. 2002; Pierce, 2005) by including studies published in the last several years and by offering suggestions for further epidemiologic research on the health of women Gulf War veterans.

## Results

Epidemiologic surveys that have examined the health of women GW veterans are summarized in Table 1. Several questions related to women’s health have been asked in surveys of the Ft. Devens, Massachusetts cohort including difficulty conceiving, whether a child was born with a birth defect, stillbirths, uterine or ovary tumors, hysterectomy, menopause, amenorrhea, vaginal yeast infections, premenstrual symptoms, pain during intercourse, difficulty achieving orgasm, and breast disease (Wolfe, et al. 2002). About 60% of the respondents met the Centers for Disease Control and Prevention (CDC) criteria for chronic multisymptom illness (CMI). In addition to female gender, positive associations were found for those with lower levels of education, self-reported use of a medical clinic in the Gulf, ingestion of anti-nerve gas pills, anthrax vaccination, tent heaters, and exposure to oil fire smoke, and chemical odors were related to CMI in logistic regression analyses.

The National Health Survey/Longitudinal Health Study of Gulf War Era Veterans (Kang, et al. 2000; Kang, et al. 2009) is one of the largest studies of the health of male and female GW and GW era veterans. A sample of 30,000 veterans (50% deployed to the Persian Gulf, 20% women) were initially invited to participate in 1995 and again in 2005 and 2012. Branch of service (Army, Navy, Air Force, and Marine Corps) and unit component (active, reserve, National Guard) were represented in both groups. A stratified random sampling method was employed to ensure that women and those who served in the reserve or National Guard were adequately represented. Although this study has provided a wealth of information about the health of GW veterans and non-deployed GW era veterans, most published analyses have controlled for gender rather than reporting gender-specific results. A notable exception is an article by Kang et al. 2005, on the role of sexual assault on the risk of PTSD among GW veterans. The adjusted odds ratio for PTSD associated with a report of sexual assault was 5.41 (95% confidence interval [CI] 3.19 – 9.17) in female veterans and 6.21 (95% CI 2.26 – 17.04) in male veterans (Kang, et al. 2005). Neither the CDC criteria for CMI nor the Kansas criteria for GWI have been used in published analyses of data from this study (Coughlin, et al. 2013). Results from the 2012 follow-up survey, which included questions about women’s health, were recently reported.

Pierce, 1997, studied a stratified representative sample of GW and GW era military service women who served in the USAF. The control group consisted of GW era women veterans who were deployed elsewhere. The study included a stratified sample of women who were interviewed about two years following the war and again in a follow-up study conducted two years later (Pierce, 1997). The sampling frame was stratified on component of the U.S. Air Force (USAF) (active, National Guard, or reserve), deployment (in the Persian Gulf theater or elsewhere), and parental status (parent or nonparent). Certain strata were over-sampled so that the sample consisted of 50% active duty, 25% reserve, and 25% guard, and 33% deployed to the theater of operations vs. 66% who served elsewhere during the same period of time. Of the 638 who were sampled, 525 (82%) women were located and, of those, 509 (97%) participated. Subsequent data collection included self-administered postal surveys that had response rates of 92% (n = 484) and 87% (n = 456), respectively (Pierce, 1997). Measures included items concerning general physical health and gender-specific health items. In addition to questions about general health status, survey items were included of 18 symptoms of gender-specific health problems and 17 medications for which medical treatment or health services were sought. Multiple statistical analyses were used to describe women’s physical and emotional health at two time points following the war. Women deployed to the theater reported significantly more general health problems as well as gender-specific health problems than did women deployed elsewhere (p < 0.05). A cluster of common health problems included: skin rash, cough, depression, unintentional weight loss, insomnia, and memory problems (Pierce, 2005). Women serving in the theater also reported a significant increase in gender-specific problems (i.e., lumps or cysts in the breasts, abnormal Pap tests) compared to women deployed elsewhere. In a further follow-up survey of this cohort of USAF women two years later, a total sample of 2,400 women were sampled (1,200 GW and 1,200 deployed elsewhere during the GW era) and 1,164 completed the survey (Pierce, 2005). About 45% of the initial sample responded. Women deployed to the theatre continued to report more health problems compared with women deployed elsewhere during the same period, after adjustment for age, education, smoking, and alcohol use (p < 0.001). An association with GW deployment was observed for 29 of 48 symptoms (Pierce, 2005).

In November 1994, the VA, DoD, and the Pennsylvania Department of Health requested that the CDC investigate a report of unexplained illnesses among members of an Air National Guard unit who were GW veterans. After an initial investigation of 59 GW veterans, which involved standardized interviews and physical examinations, a larger sample of GW veterans (n = 3, 927) were surveyed in 1995, who were members of the index unit and three comparison units in Pennsylvania and Florida (CDC, 1995; Fukuda, et al. 1998). After excluding 204 who were younger than 17 years during the GW, 1,163 (31.2%) were GW veterans and 2,560 (68.8%) had not been deployed (Fukuda, et al. 1998). In addition to general health history, the respondents were asked about the frequency, duration, and severity of 35 symptoms and possible exposures during deployment. In all units, the prevalence of each of 13 chronic symptoms was significantly greater (p < 0.05) among persons deployed to the GW than among those not deployed. The prevalence of mild-to-moderate and severe cases of CMI was 39% and 6%, respectively, among male and female GW veterans compared with 14% and 0.7% among 2,520 non-deployed veterans. Although no physical examination, laboratory, or serologic findings identified cases, veterans who met the case definition had significantly diminished functioning and well-being (Fukuda, et al. 1998). About 14% of the participants in the CDC study are women.

Unwin et al. 2002, conducted a cross-sectional mailed survey of a random sample of United Kingdom Armed Forces Personnel who were deployed to the GW, non-deployed controls and controls who were deployed to Bosnia. The GW cohort consisted of 4,000 deployed veterans plus an additional 250 women who were oversampled. The total sample size for the deployed cohort was 4,250 and the same number for the non-deployed cohort. The stratification variables were service (Royal Navy, Army, Royal Air Force), gender, age, service status (regular or reservist), rank (officer or other), and fitness (Army and Royal Air Force only). The questionnaire was completed by 645 women, 226 from the GW cohort, 227 from the Bosnia cohort, and 192 from the GW era cohort. The health of service women was compared with that of service men. The main outcome measures were physical symptoms and illnesses, functional capacity, and CMI defined using the CDC criteria. No gender differences were found for 32 of the 50 symptoms. Women were significantly more likely than men to report 6 symptoms (headaches, fatigue, constipation, stomach cramp, passing urine more often, and nausea). GW women had similar rates of ill health as their male counterparts (Unwin et al. 2002). GW women veterans were about three times as likely to meet the CDC criteria for CMI as non-deployed GW era women veterans, and as compared to women who were deployed to Bosnia (Unwin et al. 2002).

In a study conducted in Kansas in 1998, Steele, (2000); used telephone interviews of 1,548 veterans who served in the GW and 482 who served elsewhere during the GW era. All of the subjects lived in Kansas at the time of the study. In addition to general questions about military service, the respondents were asked about the severity of 37 symptoms in the past year and when the symptoms first occurred. They were also asked if they had ever been diagnosed or treated by a physician for any of 16 medical and psychiatric conditions, or for any medical condition in 5 general areas, and when each reported condition had developed. GWI, defined as having chronic symptoms in 3 of 6 domains, occurred in 34% of GW veterans, 12% of non-deployed GW era veterans who reported receiving vaccines during the war, and 4% of non-deployed GW era veterans who did not receive vaccines (Steele, 2000). The prevalence of GWI was lowest among GW veterans who served on board ship (21%) and highest among those who were in Iraq and/or Kuwait (42%). Questions about women’s health (e.g., questions to assess menopausal status) were included in the survey questionnaire. About 15.9% of the participants in the Kansas study are women. Additional analyses of data for women, and to compare female-to-male differences in risk of GWI and frequency of symptoms, are planned.

The Millennium Cohort Study (Gray, et al. 2002; Smith, et al. 2014) is one of the largest, ongoing studies of US military personnel and veterans. The study was launched in 2001 to examine deployment, demographic, behavioral, and occupational characteristics related to military service and various health outcomes. The first panel of participants invited to participate in the study was randomly selected from US military personnel who were serving in October 2000. Persons who had been deployed to Bosnia, Southwest Asia, or Kosovo between 1998 and 2000, Reserve and National Guard members, and women were oversampled. The second panel of invited participants was randomly selected from military personnel with 1 to 2 years of service as of October 2003. Marines and women were oversampled in the second panel. In an analysis of data from panels one and two (n = 73,078, 74.0% deployed to the Persian Gulf, 21.6–33.4% women), Smith et al. 2014, examined 18 symptoms that have been reported to be higher among GW veterans (severe headache; diarrhea; rash or skin ulcer; sore throat; night sweats; chest pain; unusual muscle pains; shortness of breath; trouble sleeping; unusual fatigue; sudden unexplained hair loss; being sleepy all the time; forgetfulness; stomach pain; pain in the arms, legs, or joints; cough; feeling down, depressed, or hopeless; and feeling nervous, anxious, or on edge or worrying about different things). The CDC criteria were used to assess CMI. A higher prevalence of CMI was observed among GW veterans as compared with non-deployed veterans who had served during that same era. Women had a higher prevalence of CMI over time than men (Smith, et al. 2014).

In a random sample of GW veterans from Iowa, Carney et al. 2003 compared the combat experiences, occupational and other service-related exposures, and health care use of GW male and female veterans. The sample (n = 4,886 potential subjects, 9.2% women) was stratified by GW deployment and military status (active duty, National Guard/reserve), age, gender, race, officer status, and branch of service. Deployed women (n = 129) were more often in the Army, single, without children, college educated, and reported fewer vaccinations than deployed men (n = 3,695). Men and women had similar military experiences but men more often participated in combat. Women were less likely than men to report exposures to smoke, psychological stress, and lead. No significant gender differences were found in exposure to solvents/petrochemicals, infectious diseases, neurotoxins, heat stress, trauma, or radiation (Carney, et al. 2003). Compared with male GW veterans, GW women veterans had more outpatient and inpatient health care use 5 years after deployment.

The National Survey of Women Veterans (Washington, et al. 2013) is a nationally representative sample of 3,611 women veterans surveyed by telephone in 2009. Women who served during different eras (World War II, Korea, Vietnam, first GW, and Operations Enduring Freedom, Iraqi Freedom, New Dawn) are represented. Washington et al. 2013, examined the healthcare delivery preferences and use (types of healthcare services and number of visits used, use of VA or non-VA healthcare services) of women veterans by military service era. GW era women veterans (n = 780) often cited cost of care as an important consideration.

Vogt et al. (2007; 2008) studied 495 GW veterans from across the U.S. Women were oversampled at 25%. Participants (n = 317, 26.2% women) deployed from active duty, reserve, and National Guard units and represented the Army, Navy, Air Force, Marines, and Coast Guard branches of the military. Several gender differences in exposure were observed along with gender-related differences in associations between deployment stressors and mental health outcomes. For example, as concerns about family/relationship disruptions increased, levels of anxiety increased for both women and men; however, the strength of the association was stronger for women than men. Using data from this same study, Smith et al. 2013 found that 33.8% met CDC criteria for CMI. The prevalence of CMI was not reported for men and women separately.

Several epidemiologic studies have examined reproductive outcomes among GW veterans (Cowan, et al. 1997; Araneta, et al. 2000; Kang, et al. 2001; Araneta, et al. 2003; Araneta, et al. 2004; Doyle, et al. 2004; Wells, et al. 2006; Kelsall, et al. 2007; Verret, et al. 2008). Several studies have identified significantly elevated rates of birth defects and adverse reproductive outcomes among GW veterans. This includes findings reported in 2001 from VA’s large national survey of U.S. Gulf War veterans indicating that children born to women GW veterans had nearly three times the rate of “likely” birth defects as children of women veterans of the same period who did not serve in the Gulf War (Kang, et al. 2001). In the National Health Survey (n = 15,000 GW Veterans and n = 15,000 non-deployed GW era Veterans; 20% women), Kang et al. 2001, found that male GW veterans, compared with their non-Gulf Veteran controls, reported a significantly higher rate of miscarriage (odds ratio [OR] = 1.62; 95% confidence interval [CI] = 1.32–1.99). Female GW veterans also reported more miscarriages than their respective controls, although their excess was not statistically significant (OR = 1.35; CI = 0.97–1.89). Both men and women deployed to the Gulf Theater of operations reported significant excesses of birth defects among their live born infants. These excess rates also extended to the subset of “moderate to severe” birth defects [males: OR = 1.78 (CI = 1.19–2.66); females: OR = 2.80 (CI = 1.26–6.25)]. No statistically significant differences by deployment status were found among men or women for stillbirths, preterm deliveries or infant mortality (Kang, et al. 2001). Overall, however, findings have varied with different study designs and sample sizes, with some studies showing elevated risks of stillbirths, miscarriages, and/or birth defects (Kang, et al. 2001; Araneta, et al. 2003; Araneta, et al. 2004). Increased risks of ectopic pregnancies and spontaneous abortions have been observed in some studies (Araneta, et al. 2000; Araneta, et al. 2004).

## Discussion

The results of this review indicate that there is currently a paucity of epidemiologic studies that have evaluated GWI and other high interest health outcomes in women veteran subgroups, e.g., subgroups identified by deployment characteristics (e.g. location, exposures, and branch of service). Studies often show differences, however, there are limitations in the research that need addressing, including limited numbers of women and variability in job and deployment times. Some, but not all studies adjusted for mental health conditions such as post-traumatic stress disorder when looking at physical health conditions. There may also have been residual confounding or unmeasured confounders in studies that compared male and female Gulf War veterans or in studies that compared Gulf War women veterans and controls. There is a need for additional studies of women veterans who served in the 1990–1991 Gulf War and comparison groups of women who served in other locations during that same time period. This should include extended analyses of existing datasets and new data collection. Much more is known now about the complex configuration of symptoms reported at the time of the 1990–1991 Gulf War. Current, comprehensive data are needed on the health status of women who served during the 1990–1991 Gulf War, and appropriate controls, in order to identify specific conditions that affect GW women veterans at excess rates. Some medical conditions (e.g., rheumatoid arthritis, chronic fatigue syndrome, migraine headache) are known to be more common among women than men, both among veteran and non-veteran populations. Among the questions of interest are: what is the prevalence of GWI among women GW veterans, defined by both the CDC and Kansas criteria (Fukuda, et al. 1998; Steele, 2000). What is the frequency and patterns of veteran-reported chronic symptoms and medical conditions diagnosed by healthcare providers? What is the prevalence of female-specific health symptoms and medical conditions? What are their general health and functional status and use of healthcare services including hospitalizations? Studies are needed to examine sex-differences in GWI including female-to-male differences in the frequency of symptoms that are associated with GWI and the overall-all prevalence of GWI among GW female and male veterans. In particular, longitudinal studies are needed to help separate out exposure outcomes from “normal” aging processes that occur over time. There is a need for longitudinal assessments of changes in GW era women veterans’ health over time, using baseline data collected in the original population studies from which current cohort samples are drawn. Studies are also needed to provide comprehensive data on veteran-reported pregnancy and birth outcomes among GW and GW era women veterans.

Few epidemiologic studies that have evaluated GWI and other high interest health outcomes in women veteran subgroups, by subgroups identified by age and menopausal status (e.g., pre-, peri- and post-menopause subgroups). Theories about the pathobiology of GWI focus on neuro-immune mechanisms and those mechanisms may be altered by menopause. Menopause has effects on a number of organ systems including the cardiovascular, skeletal, central nervous and genitourinary systems (Gameiro, et al. 2010; Sammaritano, 2012). Studies have shown that following menopause, women can experience an increase in pro-inflammatory serum markers such as IL-1, IL-6, and TNF-alpha (Gameiro, et al. 2010). Given that many women veterans who were deployed to the Gulf region may be in the midst of natural or surgically induced menopause, more research on this topic is important.

There remains a need to evaluate birth outcomes in appropriate subgroups, e.g., by time period of birth, by parental exposures and by other deployment characteristics (e.g., whether the women were later deployed to Operation Enduring Freedom or Operation Iraqi Freedom). There is a paucity of subgroup analyses assessing rates of birth defects and other reproductive outcomes by time period of birth and by parental exposures and other deployment characteristics. Although data from a large GW birth defect registry suggests there may have been significant increases in rates of some birth defects in the early years after the war, but that those differences leveled off over time, GW birth defect studies have not compared rates in GW vs. non deployed GW era veterans by birth year/time period, or by whether the parent veteran had GWI or experienced exposures that may be associated with birth outcomes. Higher diagnosis of birth defects and adverse reproductive outcomes in Gulf War women veterans compared to controls might be in part due to higher awareness, concerns about health issues related to the Gulf War, better access to health care and differential quality of care that veterans receive. Further research is needed to address these important issues.

In summary, further research is needed to provide a comprehensive picture of the health of women GW veterans and to examine a broad range of women’s health issues including adverse reproductive outcomes (spontaneous abortions, still births, ectopic pregnancies, pre-term births, and birth defects). This includes assessments of current health status, changes in health symptoms and conditions over time, and possible differences in health outcomes associated with specific experiences and exposures during the war. Future studies would be strengthened by assessing GWI symptom patterns that may be specific to women veterans, examine diagnosed medical conditions among women veterans, and evaluate changes in women’s health over time, including changes potentially associated with menopause and age. Such data would improve our understanding of GWI in women veterans who served in the Gulf War, women GW veteran’s health, and adverse reproductive outcomes, and lay the groundwork for future research aimed at a short-term or longer-term improvement in clinical treatment of women veterans with GWI, and the definition and diagnosis of GWI in women.

## Figures and Tables

**Table 1:** Design characteristics of studies of U.S. veterans of the first Gulf War that provided information about the health of women veterans.

Study	Inception	Design	Administration	Population eligible to participate	Focus
Devens Cohort Study (Wolfe et al. 2002)	1991, 1995–1996, 1997, 2013 (ongoing)	Repeat cross-sectional surveys of established cohort	Initial re-adjustment survey, with follow-up surveys by mail or in-person	Deployed Army soldiers (84 units, n = 2,300) returning from GW through Ft. Devens, MA	Physical and psychological health, symptoms, reproductive history, adverse birth outcomes, women’s health, domestic and military exposures
Longitudinal Health Study (Kang et al. 2000, 2009)	1993–1995	Cross-sectional survey of established cohort with follow-up surveys conducted in 2005 and 2012	Mail survey, telephone interviews; web-based survey added in 2012	National sample of GW (n=15,000) and GW era (n=15,000) veterans	Health status, health care use, physical and psychological health conditions, symptoms, military exposures. Questions on reproductive health outcomes were included in the 1995 survey. Questions on women’s health were included in the 2012 follow-up survey
USAF (Pierce 1997, 2005)	1993	Cross-sectional survey and follow-up surveys conducted 2 and 4 years later	Mail surveys	Sample of 525 USAF women (expanded to 2,400 women for the second follow-up survey) who served in USAF, stratified on component (active, National Guard, or reserve), deployment (in the theater or deployed elsewhere), and parental status (parent or nonparent)	Physical and psychological health, symptoms, gender-specific health
Air Nat’l Guard (Fukuda et al. 1998; CDC 1995)	1994	Cross-sectional		GW veterans from a PA-based Air National Guard unit, two USAF reserve units (PA, FL) and an active duty USAF unit (FL) (n=3,927)	Physical health, symptoms, risk factors for illness
(Unwin et al. 2002)	Post-1997	Cross-sectional survey	Mail survey	Random sample of UK Armed Forces Personnel. 4,250 deployed to GW, 4,250 non-deployed, plus Bosnia cohort. Women (n=1,026) were over-sampled	Physical and psychological symptoms, medical disorders, military exposures
Kansas (Steele 2000)	1998	Cross-sectional study	Telephone survey	KS veterans or reserve members (n=2,030) who served on active duty between 8/90 and 7/91	Physical and psychological health, symptoms, military exposures
Millennium Cohort Study (Gray et al. 2002; Smith et al. 2014)	2001	Repeated cross-sectional surveys	Mail survey, telephone survey	Random sample of US military personnel serving in October 2000 (panel one). Random sample of military personnel with 1 to 2 years of service as of October 2003 (panel two), and more recent panels. Marines and women were over sampled in panel two	Health status, health care use, medical and psychological health, symptoms, military exposures
Iowa Gulf War Study (Carney et al. 2003)	1995–1996	Cross-sectional study	Telephone survey	Sample of 4,886 GW era veterans from Iowa (9.2% women) stratified on GW deployment and military status, age, gender, race, officer status, and branch of service	Health status, health care use, military preparedness and exposures
National Survey of Women Veterans (Washington et al. 2013)	2009	Cross-sectional survey of random sample of women veterans	Telephone survey	National sample of 3,611 women veterans	Healthcare delivery preferences, health care use, general health status, physical and psychological health conditions
Survey of GW veterans (Vogt et al. 2007, 2008; Smith et al. 2013)	Not stated	Cross sectional survey	mail survey	National sample of 495 GW veterans (25% women) from all branches of service and unit components	Combat experiences, perceived threat, difficult living and working environment, concerns about family/relationship disruptions, sexual harassment, psychological health conditions, physical symptoms

**Table 2:** Participant characteristics and findings of studies of U.S. veterans of the first Gulf War that provided information about the health of women veterans.

Study	Number of GW women who participated	Number of GW men who participated	Comparison group	Findings	Other Information
Devens Cohort Study (Wolfe et al. 2002)	Of 1,290 participants in the 1997 survey, 10.4% were women GW veterans	In 1997, 90.6% of 1,290 respondents were men	N/A	Female gender, lower levels of education, self-reported use of a medical clinic in the Gulf, ingestion of anti-nerve gas pills, anthrax vaccination, tent heaters, and exposure to oil fire smoke, and chemical odors were related to MSI in logistic regression analyses.	Additional analyses of data for women are planned.
National Health Study/Longitudinal Health Study (Kang et al. 2000, 2005, 2009)	11,441 GW veterans, 19.7% women	9,476 non-deployed GW era veterans		Among GW veterans, the adjusted odds ratio for PTSD associated with a report of sexual assault was 5.41 (95% confidence interval [CI] 3.19–9.17) in female veterans and 6.21 (95% CI 2.26–17.04) in male veterans (19).	
USAF (Pierce 1997, 2005)	160 to 625	N/A	365 to 539 GW era women deployed elsewhere		Women deployed to the Gulf reported increased general health problems, skin rash, cough, depression, unintentional weight loss, insomnia, memory problems, breast lumps or cysts, and abnormal Pap tests than did women deployed elsewhere (p<0.05).
Air Nat’l Guard (Fukuda et al. 1998; CDC 1995)	Of 3,723 participants, 14% were female, 47% were deployed to the Persian Gulf. The number of deployed women was not reported	About 86% were male, 47% were deployed; the number of deployed men was not reported	Air Nat’l Guard not deployed to the Persian Gulf		Additional analyses of data for women are planned.
(Unwin et al. 2002)	226 GW women		192 non-deployed GW era women, and 227 deployed to Bosnia	Women were significantly more likely than men to report 6 symptoms (headaches, fatigue, constipation, stomach cramp, passing urine more often, and nausea). Women deployed to the Persian Gulf had similar rates of ill health as their male counterparts. GW women veterans were about three times as likely to meet the CDC criteria for CMI than GW era women veterans, and as compared to women who were deployed to Bosnia.	
Kansas (Steele 2000)	216	1,331	482 non-deployed GW era veterans residing in KS		Questions about women’s health (e.g., questions to assess menopausal status) were included in the survey questionnaire.
Millennium Cohort Study (Smith et al. 2014)	n=73,078, 74.0% deployed to the Persian Gulf, 21.6–33.4% women	n=73,078, 74.0% deployed to the Persian Gulf, 66.6–78.4% men		A higher prevalence of CMI was observed among GW veterans as compared with non-deployed veterans who had served during that same era. Women had a higher prevalence of CMI over time than men	
Iowa Gulf War Study (Carney et al. 2003)	129 GW women	3,695 GW men	206 non-deployed GW era women	Men and women had similar military experiences but men more often participated in combat. Men were more likely than women to report exposures to smoke, psychological stress, and lead. No significant gender differences were found in exposure to solvents/petrochemicals, infectious diseases, neurotoxins, heat stress, trauma, or radiation. Compared with male GW veterans, GW women veterans had more outpatient and inpatient health care use 5 years after deployment.	
National Survey of Women Veterans (Washington et al. 2013)	780 GW era women veterans, deployment status not reported	N/A	WW II, Korea era, Vietnam era, and OEF/OIF/OND era women veterans	GW era women veterans (n=780) often cited cost of care as an important consideration.	
Survey of GW veterans (Vogt et al. 2007, 2008; Smith et al. 2013)	83 GW women	234 GW men		Several gender differences in exposure were observed along with gender-related differences in associations between deployment stressors and mental health outcomes. Among men and women combined, 33.8% met CDC criteria for CMI.	
